# Complete Circular Genome Sequence of a *mecB-* and *mecD*-Containing Strain of Macrococcus canis

**DOI:** 10.1128/MRA.00408-21

**Published:** 2021-06-03

**Authors:** Sybille Schwendener, Vincent Perreten

**Affiliations:** a Institute of Veterinary Bacteriology, Vetsuisse Faculty, University of Bern, Bern, Switzerland; Montana State University

## Abstract

The complete genome sequence of Macrococcus canis strain 19/EPI0118, isolated from a veterinary clinic environment in Switzerland, was determined using hybrid assembly of Oxford Nanopore and Illumina reads. 19/EPI0118 harbored the methicillin resistance genes *mecB* and *mecD* on a staphylococcal cassette chromosome *mec* element and a *Macrococcus* chromosomal resistance island, respectively.

## ANNOUNCEMENT

Macrococcus canis is a Gram-positive bacterium which has been found on the skin of healthy dogs and in infection sites (1, 2). Some *M. canis* strains carry the methicillin resistance gene *mecB* on staphylococcal cassette chromosome *mec* (SCC*mec*) elements or on a plasmid (3, 4). The *mecD* gene has so far only been associated with chromosomal resistance islands McRI*_mecD_* in Macrococcus caseolyticus and an SCC*mec* element in Macrococcus bohemicus (5–7). Recently, a methicillin-resistant *M. canis* strain (19/EPI0118) isolated from the environment of a companion animal clinic in Switzerland was found to contain both the *mecB* and *mecD* genes (8). The strain was also resistant to fusidic acid (8). We report here the complete genome sequence of 19/EPI0118 and the genetic elements containing *mecB*, *mecD*, and *fusC*.

Genomic DNA of 19/EPI0118 (cryopreserved in our collection) was extracted from culture grown overnight at 37°C on Trypticase soy agar containing 5% sheep blood, using the MasterPure complete DNA and RNA purification kit (Lucigen, Middleton, WI). DNA sequencing was performed using a NEBNext Ultra II directional DNA library with TruSeq adapters on an Illumina NovaSeq 6000 system (2 × 150-bp paired-end reads) at Eurofins Genomics (Germany) and in-house with a MinION system (Oxford Nanopore Technologies [ONT]; R9.4.1 SpotON flow cell, MinION MK1b device). The ONT library was prepared using the 1D ligation sequencing kit (SQK-LSK109) and the native barcoding expansion kit (EXP-NBD104) (Oxford Nanopore Technologies, UK). The ONT reads were base called and demultiplexed using Guppy software v4.4.1 and end trimmed and size filtered using Cutadapt v2.5. The Illumina reads were used without filtering after confirmation of high-quality and adaptor-free sequences by FastQC v0.11.7 analysis. The genome sequence of 19/EPI0118 was *de novo* assembled and circularized using Unicycler v0.4.8 run with default parameters, using paired-end Illumina reads (8,580,460 reads, 1,287,069,000 total bases, 543× coverage) and ONT reads larger than 10 kb (22,513 reads; mean, 20,422 bp; 460,021,332 total bases; 194× coverage) (9). All calculations were performed on the high-performance computing (HPC) cluster UBELIX at the University of Bern (http://www.id.unibe.ch/hpc). The genome was annotated using the NCBI Prokaryotic Genome Annotation Pipeline service (10).

**FIG 1:**
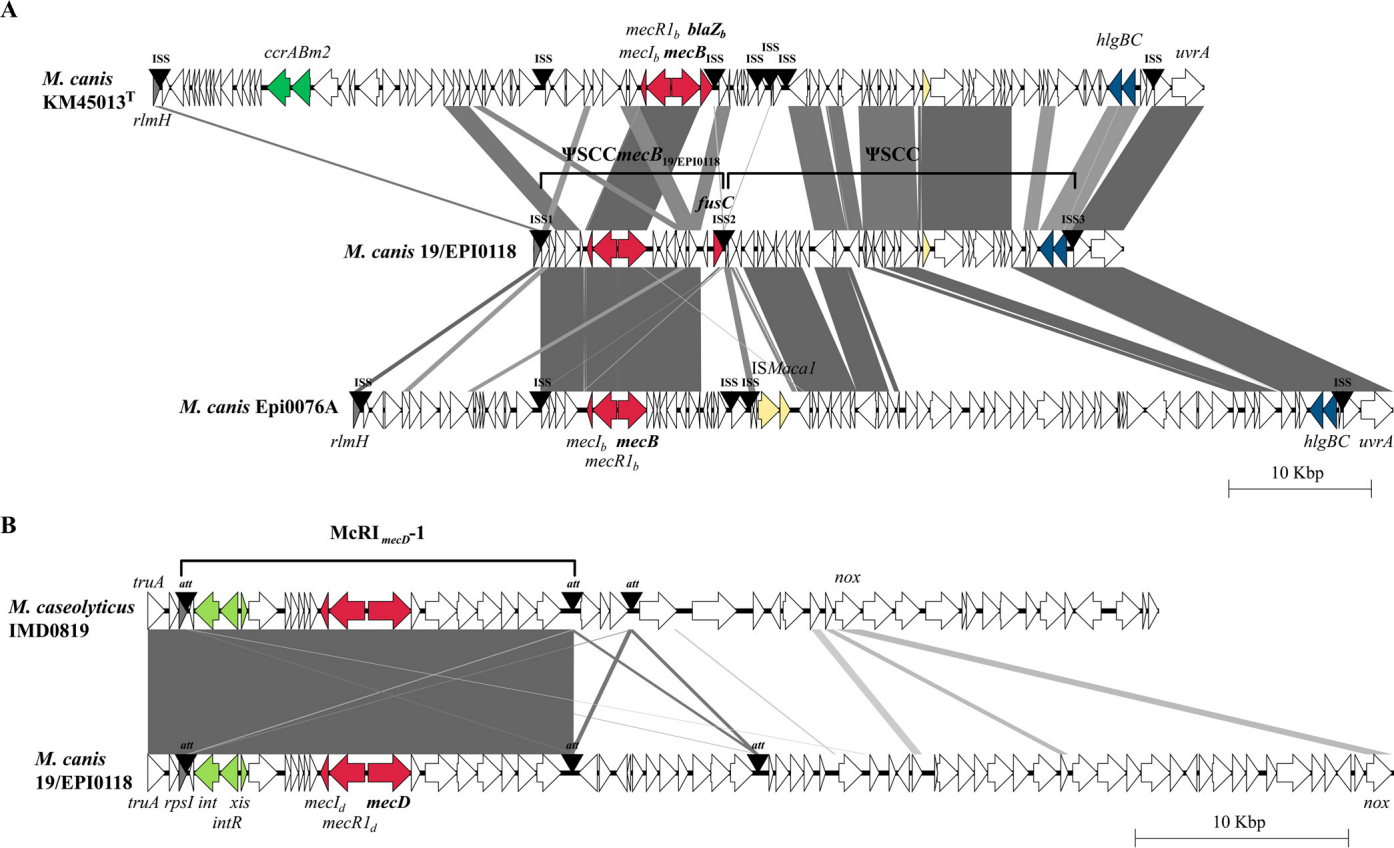
The genetic structure of ΨSCC*mecB*_19/EPI0118_ and McRI*_mecD_*-1 elements in *M. canis* 19/EPI0118. (A) (Ψ)SCC*mecB* elements in *M. canis*. Chromosomal regions from the 23S rRNA (pseudouridine[1915]-*N*[3])-methyltransferase gene (*rlmH*) to the UvrABC system protein A gene (*uvrA*) were compared for *M. canis* strains KM45013^T^ (GenBank accession number CP021059), 19/EPI0118 (CP072837), and Epi0076A (CP047363). The *rlmH* gene (gray) contains at the 3′ end the integration site sequence (ISS) for SCC. Further copies of ISS that subdivide composite (Ψ)SCC*mec* elements are indicated. The ISS have the following sequences for 19/EPI0118: ISS1, 5′-GAAAGTTATCATAAATGA; ISS2, 5′-GAAAGTTATCATAAGTGA; ISS3, 5′-TGGGTATATCAAAAATAA; they flank the ΨSCC*mecB*_19/EPI0118_ (CP072837, positions 32,310 to 45,199) and ΨSCC (CP072837, positions 45,200 to 69,684). (B) Comparison of McRI*_mecD_*-1 of *M. canis* 19/EPI0118 (CP072837, positions 259,551 to 277,684) with McRI*_mecD_*-1 of *M. caseolyticus* IMD0819 (CP021058). The chromosomal region downstream of the tRNA pseudouridine(38-40) synthase gene (*truA*) was compared. The attachment site (*att*) for McRI*_mecD_* at the 3′ end of the 30S ribosomal protein S9 gene (*rpsI*) (gray) and copies of it that delimitate chromosomal islands are indicated. The core *att* sequence is 5′-GAACGTAA(A/G)AA(A/G)CCAGGTCTTAAAGGCGCTCGTCGTTCACCACAGTTCTCAAAACGTTAAT (positions containing an A or G base are given in parenthesis). Genes are represented by arrows and are color-coded as follows: red, *mec* operon genes and fusidic acid resistance gene; green, *ccr* genes; light green, integrase (*int*) and associated genes of McRI*_mecD_*; yellow, other recombinase genes; blue, putative hemolysin genes (*hlgBC*). Figures were generated using Easyfig software (11). Gray connections indicate regions with between 72% and 100% nucleotide sequence identity.

The complete genome of 19/EPI0118 contained a circular chromosome of 2,359,135 bp and four small circular plasmids (3,561, 2,426, 1,888, and 1,436 bp) with a GC content of 36.5%. A total of 2,362 protein-coding sequences and 78 RNAs, including 58 tRNAs and 5 16S-23S rRNA clusters, were predicted. The *mecB* gene was found on a pseudo (Ψ) SCC*mec* (ΨSCC*mecB*_19/EPI0118_) integrated at the 3′ end of the chromosomal *rlmH* gene, followed by a ΨSCC (Fig. 1A). Cassette chromosome recombinase (*ccr*) genes were not present, and the *mecB* operon lacked the β-lactamase *blaZ_m_* gene. The fusidic acid resistance gene *fusC* was found at the 3′ end of ΨSCC*mecB*_19/EPI0118_ (Fig. 1A). The *mecD* gene was part of an McRI*_mecD_*-1 integrated at the 3′ end of the *rpsI* gene (Fig. 1B). This element was, except for three mismatches, identical to the McRI*_mecD_*-1 of *M. caseolyticus* IMD0819 (5).

These findings showed that a *Macrococcus* strain can carry two methicillin resistance genes in its chromosome and suggests exchange of McRI*_mecD_* between *M. canis* and *M. caseolyticus*.

### Data availability.

The complete genome sequence of *M. canis* strain 19/EPI0118 has been deposited in GenBank under accession numbers CP072837, CP072838, CP072839, CP072840, and CP072841. The associated BioProject and BioSample accession numbers are PRJNA720016 and SAMN18630837, respectively. The raw reads were deposited in the SRA database with accession numbers SRR14161194 (Illumina) and SRR14161193 (ONT).
